# Rett syndrome: a neurological disorder with metabolic components

**DOI:** 10.1098/rsob.170216

**Published:** 2018-02-14

**Authors:** Stephanie M. Kyle, Neeti Vashi, Monica J. Justice

**Affiliations:** 1 Genetics and Genome Biology Program, The Hospital for Sick Children, The Peter Gilgan Centre for Research and Learning, Toronto, Ontario, Canada M5G 0A4; 2 Department of Molecular and Human Genetics, Baylor College of Medicine, Houston, TX 77030, USA; 3 Department of Molecular Genetics, University of Toronto, Toronto, Ontario, Canada M5S 1A1

**Keywords:** Rett syndrome, methyl-CpG-binding protein 2, histone deacetylase, nuclear corepressor, metabolism

## Abstract

Rett syndrome (RTT) is a neurological disorder caused by mutations in the X-linked gene methyl-CpG-binding protein 2 (*MECP2*), a ubiquitously expressed transcriptional regulator. Despite remarkable scientific progress since its discovery, the mechanism by which *MECP2* mutations cause RTT symptoms is largely unknown. Consequently, treatment options for patients are currently limited and centred on symptom relief. Thought to be an entirely neurological disorder, RTT research has focused on the role of *MECP2* in the central nervous system. However, the variety of phenotypes identified in *Mecp2* mutant mouse models and RTT patients implicate important roles for MeCP2 in peripheral systems. Here, we review the history of RTT, highlighting breakthroughs in the field that have led us to present day. We explore the current evidence supporting metabolic dysfunction as a component of RTT, presenting recent studies that have revealed perturbed lipid metabolism in the brain and peripheral tissues of mouse models and patients. Such findings may have an impact on the quality of life of RTT patients as both dietary and drug intervention can alter lipid metabolism. Ultimately, we conclude that a thorough knowledge of MeCP2's varied functional targets in the brain and body will be required to treat this complex syndrome.

## 1. Rett syndrome: clinical features and stages

Rett syndrome (RTT, OMIM #312750) was first described by Andreas Rett, an Austrian paediatric neurologist, after observing two female patients with identical hand-wringing stereotypies in his clinic waiting room. Upon examination, he found that both patients had the same history: normal early development, followed by a period of regression and loss of purposeful hand movements. Intrigued, Dr Rett documented other female patients in his clinic with similar symptoms. Believing the symptoms to be consistent with a metabolic disorder, he called it ‘cerebroatrophic hyperammonaemia’ in a 1966 German publication [1]. However, the disorder did not gain general acceptance among the medical community until its description in English publications 17 years later [2]. RTT is now well known as a progressive neurological disorder that primarily affects girls, occurring in 1 : 10 000–15 000 live female births [3].

**Figure 1.**
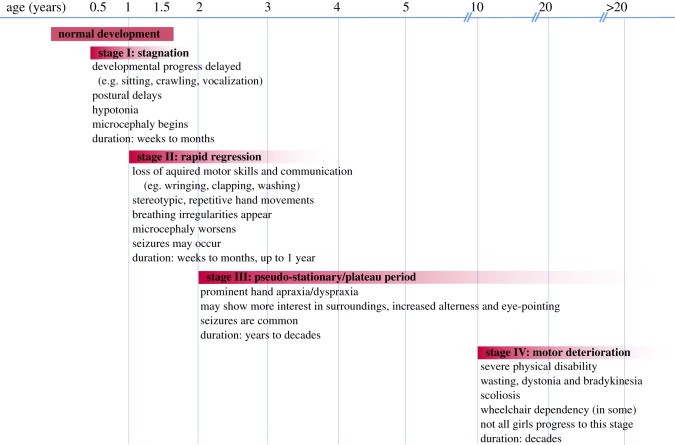
Timeline of stages and symptom onset in RTT patients. Rett syndrome (RTT) is divided into four progressive stages. Patients display seemingly normal early development. Between 6 and 18 months of age, patients experience a period of developmental stagnation (Stage I) and no longer meet their mental, cognitive or motor milestones. Head circumference growth slows and this period lasts for weeks to months. Stage II is defined by rapid developmental regression in which acquired purposeful hand movements and verbal skills are lost. Microcephaly worsens and breathing irregularities and/or seizures arise. Stage III is a pseudo-stationary plateau period in which patients may show mild recovery in cognitive interests, but purposeful hand and body movements remain severely diminished. Stage IV is defined by motor deterioration and may last decades. Many patients are wheelchair and/or gastrostomy-tube dependent. However, not all girls progress to this severe stage.

The clinical diagnosis of RTT is based on a battery of co-existing and well-defined inclusive and exclusive criteria (summarized in [4–6]). Following a period of normal neurological and physical development during the first 6–18 months of life, the first features of RTT begin to manifest in early childhood and appear progressively over several stages (figure 1): stagnation (age 6–18 months), rapid regression (age 1–4 years), pseudostationary (age 2–potentially life) and late motor deterioration (age 10–life). Characteristic symptoms of RTT include loss of acquired speech and motor skills, repetitive hand movements, breathing irregularities and seizures. RTT patients may also suffer from sporadic episodes of gastrointestinal problems, hypoplasia, early-onset osteoporosis, bruxism and screaming spells [4]. Despite these impairments, RTT patients are well integrated into their families and enjoy personal contact [7]. The development of new augmentative communication technologies has allowed otherwise non-verbal RTT patients to engage with others and express themselves [8].

Many children diagnosed with RTT have reduced brain volume compared with healthy individuals, consistent with a smaller head circumference [9,10]. Reduced brain volume is largely due to small neuronal body size and a denser packing of cells, particularly in layers III and V of the cerebral cortex, thalamus, substantia nigra, basal ganglia, amygdala, cerebellum and hippocampus [11]. Patients also have reduced dendritic arborization, indicative of a delay in neuronal maturation [12]. Furthermore, hypopigmentation of the substantia nigra suggests a dysfunction of dopaminergic neurons [9]. RTT patients show evidence of dysregulated neurotransmitters, neuromodulators and transporters, indicating an important role in synaptic function [13,14].

Metabolic complications are also common in RTT. A number of patients present with dyslipidaemia [15,16], elevated plasma leptin and adiponectin [17,18], elevated ammonia [1] and inflammation of the gallbladder, an organ which stores bile for fat digestion [19]. Changes in brain carbohydrate metabolism [20] and neurometabolites associated with cell integrity and membrane turnover [21,22] have also been reported. Additionally, energy-producing mitochondria have abnormal structure in patient cells [23–26]. Consistently, altered electron transport chain complex function [27], increased oxidative stress [28–30], and elevated levels of lactate and pyruvate in blood and cerebrospinal fluid [20,27] have been observed in RTT patients (reviewed in [31]).

Treatment for RTT patients is currently limited to symptom control. With adequate attention to orthopaedic complications, seizure control and nutrition, women with RTT may survive into middle age and older. However, patients have a sudden and unexpected death rate of 26%, much higher than healthy individuals of a similar age, and typically die due to respiratory infection, cardiac instability and respiratory failure [32–34].

## 2. Mutations in *MECP2* cause Rett syndrome

Familial cases were instrumental in determining the genetic cause of typical RTT. Multipoint linkage analysis of a Brazilian RTT family with three affected and three unaffected daughters narrowed the location of the gene to Xq28 [35]. Following this breakthrough in 1999, Amir *et al*. [36] systematically analysed nearly 100 candidate genes located in the Xq28 region for mutations in RTT patients. By screening genomic DNA from sporadic and familial RTT patients, the group identified damaging missense, frameshift and nonsense mutations in the coding region of the gene methyl-CpG-binding protein 2 (*MECP2*) in seven patients. It is now well established that mutations in *MECP2* account for 95% of typical RTT cases and commonly occur *de novo* [36–38]. RTT patients are heterozygous for *MECP2* mutation, carrying one normal and one mutated copy of *MECP2.* When a child presents with RTT-like symptoms, but does not fulfil all the diagnostic criteria for RTT, they may be diagnosed with atypical RTT, symptoms of which deviate in age of onset, sequence of clinical profile and/or severity (table 1) [39–49]. Many atypical cases are associated with mutations in X-linked cyclin-dependent kinase-like 5 (*CDKL5*; OMIM #300203) or Forkhead box G1 (*FOXG1*; OMIM #164874), but some remain undefined [39–49]. Mutations in *MECP2* have also been associated with intellectual disability, autism and lupus erythematosis [50,51].

**Table 1.** Atypical Rett syndrome variants. These variants may be milder or more severe than classical RTT symptoms.

type	description
**severe atypical RTT variants**	
early-onset seizure type	— can be caused by mutation in X-linked cyclin-dependent kinase-like five gene (CDKL5; OMIM #300203) — seizures in the first months of life — develop RTT symptoms
congenital variant	— can be caused by mutation in the Forkhead box G1 (FOXG1; OMIM #164874) gene located on chromosome 14 — born with congenital microcephaly and intellectual disability — lack of normal psychomotor development — develop RTT symptoms during first three months of life
**milder atypical RTT variants**	
late regression type	— develop RTT symptoms at a preschool age
preserved speech ‘Zapella’ type	— develop RTT symptoms but recover some verbal skills and can form phrases and sentences
‘Forme fruste’ variant	— the most common atypical variant accounting for 80% of cases well-preserved motor skills and only subtle neurological abnormalities such as mild hand dyspraxia

MeCP2 is an abundant nuclear protein that was originally identified in a screen for proteins with methyl-DNA-specific binding activity. It is ubiquitously expressed throughout all human tissues, but is particularly abundant within neurons [52,53]. In the central nervous system (CNS), MeCP2 is expressed at low levels prenatally, but progressively increases during neuronal maturation and synaptogenesis reaching its peak in mature, post-migratory neurons, suggesting a role for MeCP2 in maintaining neuronal maturation, activity and plasticity [54–57].

**Figure 2.**
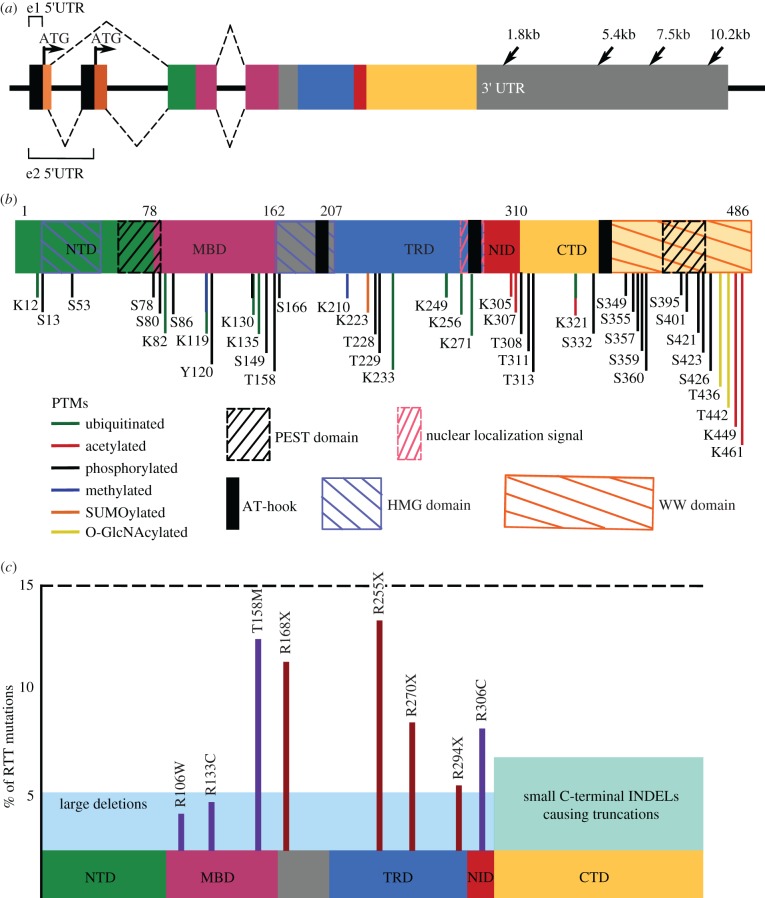
Mutations in the multifunctional protein MeCP2 cause RTT. Coloured boxes indicate different encoded functional domains: light orange, N-terminus of MeCP2_e1; dark orange, N-terminus of MeCP2_e2; green, N-terminal domain (NTD) which has identical amino acid sequences between the two isoforms; pink, methyl-binding domain (MBD); blue, transcriptional repression domain (TRD); red, nuclear coreceptor co-repressor (NCoR) interaction domain (NID); yellow, C-terminal domain (CTD). (*a*) The four exons in the *MECP2* gene. Arrows in exons 1 and 2 indicate the ‘ATG’ start codons for MeCP2_e1 or MeCP2_e2, respectively. Arrows in the 3′ UTR indicate multiple polyA sites resulting in different-length transcripts. Dashed lines on the top indicate the splicing pattern of MeCP2_e1 and dashed lines on the bottom indicate the splicing pattern of MeCP2_e2. (*b*) Functional domains and post-translational modifications (PTMs) of MeCP2. Coordinates are in relation to isoform MeCP2_e2. MeCP2 contains two PEST domains (black slashed boxes), two HMG domains (blue slashed boxes), three AT-hook domains (black solid boxes), one functional nuclear localization signal (NLS) (pink slashed box) and one WW domain (orange slashed box). PTMs are scattered throughout the protein and regulate interactions with MeCP2 binding partners. (*c*) Common damaging *MECP2* mutations. Schematic of MeCP2 with functional domains. *y*-axis represents percentage of RTT patients with indicated mutation. Missense mutations are in purple and nonsense mutations are in red. Combined, these point mutations make up approximately 70% of all RTT-causing mutations.

*MECP2* is encoded by four exons which are expressed as the MeCP2_exon1 (e1) and the MeCP2_exon2 (e2) isoforms (figure 2*a*) [57–59]. MeCP2_e1 is 10 times more abundant than MeCP2_e2 in human and mouse brain and in mouse thymus and lung, whereas a 1 : 1 ratio is seen in mouse testis and liver [60,61], suggesting that e1 is the primary functional isoform in the brain. Exon 4 in both mouse and human contains an 8.5 kb 3′UTR, which is one of the longest known in the human genome, and features four polyadenylation (polyA) sites resulting in four differentially expressed transcripts (figure 2*a*) [62–64]. The 3′UTR may regulate downstream translation of *MECP2*'s transcript by controlling mRNA degradation and stability, nucleocytoplasmic transport, mRNA localization and modulation of translation [65]. The alternate *MECP2* transcripts show quantitative differences in expression in different tissues, in different stages of mouse embryonic development and in human post-natal brain development [62].

MeCP2 consists of four primary functional domains (figure 2*b*). The binding specificity of MeCP2 is dependent on the presence of methylated DNA and its methyl-binding domain (MBD) located at amino acids 78–162 [53,66]. MeCP2 is different from other methyl-DNA binding proteins because of its ability to interact with a single, symmetrical methylated CpG (mCpG) site [53,66]. However, recent work by Lagger *et al.* revealed that MeCP2 also has a high binding affinity for methylated and hydroxymethylated CAC (mCAC and hmCAC, respectively) in neurons, demonstrating that both methylated dinucleotides and trinucleotides can recruit MeCP2 to DNA for transcriptional regulation [67]. A transcriptional repressor domain (TRD) occurs in amino acids 207–310, and contains the NCoR-interaction domain (NID), which facilitates binding of MeCP2 to the NCoR1/SMRT co-repressor complex [68–71]. The C-terminal domain (CTD) also shows DNA-binding ability, suggesting the presence of a chromatin-interacting domain in the vicinity of the 5′ region of the CTD [72].

CpG methylation is minimal in invertebrate genomes (10–40%), but very high in vertebrate genomes (60–90%) [73,74]. Methylated DNA sites recruit methyl-DNA-binding proteins like MeCP2, which attract transcriptional regulatory complexes [75]. Consistently, MeCP2 represses transcription in a gene-specific manner, interacting with the co-repressor complexes mSIN3A and NCoR1/SMRT [70,76]. However, MeCP2 may also facilitate transcriptional activation [77], chromatin compaction [78,79] and mRNA splicing [80,81], while also interacting with a host of other regulatory proteins involved in a variety of molecular pathways [81–84].

Despite having well-defined functional domains, MeCP2 classifies as an intrinsically disordered protein (IDP) and acquires tertiary structure upon interaction with other protein partners or nucleic acids [85]. Disordered structure makes IDPs notoriously promiscuous binding partners [86]. As such, post-translational modifications (PTMs) add an additional layer of regulation to ensure MeCP2 binding to appropriate partners. Accordingly, MeCP2 undergoes many PTMs including acetylation, phosphorylation, ubiquitination and SUMOylation (figure 2*b*; reviewed in [87,88]), which strongly regulate interactions. Altogether, multiple studies suggest that genes can be activated or repressed by MeCP2, depending upon the cellular context [77,89,90], suggesting that MeCP2 may be defined as a transcriptional modulator.

## 3. Phenotype variation among people with *MECP2* mutations

According to the Human Gene Mutation database, 555 RTT-causing mutations have been identified in *MECP2* [91]*. De novo* mutations account for 99.5% of the mutations in *MECP2*, of which approximately 70% are C > T transitions, which typically arise due to hypermutability at mCpG dinucleotides within the *MECP2* locus [48]. Unlike the X chromosome in oocytes, the X chromosome in sperm is hypermethylated. It has been speculated that spontaneous deamination of methylated cytosine residues may result in a transition to a thymine, increasing the potential for deleterious mutations at hypermethylated CpG sites [92,93]. As such, most *de novo MECP2* mutations originate from the paternally inherited X chromosome [94].

The wide spectrum of *MECP2* mutations includes point mutations, insertions, duplications, small or large deletions, or whole *MECP2* gene deletions. Despite this, only eight missense and nonsense mutations (R106 W, R133C, T158M, R168X, R255X, R270X, R294X and R306C) account for approximately 70% of all mutations in RTT (figure 2*c*). C-terminal deletions account for another approximately 8%, and large deletions constitute approximately 5% [38]. Mutation types tend to cluster: missense mutations frequently occur in the MBD, while nonsense mutations generally occur downstream of the MBD [38]. Frameshift mutations resulting from small deletions usually occur in the C-terminus (figure 2*c*) [48]. The location of the mutation reduces specific aspects of MeCP2 function [95]. For example, mutations located in the MBD reduce the DNA-binding ability of MeCP2.

In early studies, Hagberg *et al*. [2] noted that the severity of motor impairment in classic female RTT patients ranged from wheelchair bound before age five to retaining the ability to walk with a Parkinsonian-like gait. Given the wide variety of mutation types and phenotype severity, genotype–phenotype studies have correlated mutation status with clinical features of RTT patients [38]. Early truncating mutations in the *MECP2* gene such as R168X, R255X and R270X, and large INDELs, cause the most severe phenotype. Missense mutations such as R133C and R306C, late truncating mutations such as those in R294X, and others in the 3′ end, which keep the MBD and most of the TRD intact, are the mildest [38,96]. Therefore, *MECP2* mutation status is a strong predictor of disease severity, but phenotype variation commonly occurs between individuals with the same *MECP2* mutation and is attributed to differences in X chromosome inactivation (XCI) [97].

Female heterozygous RTT patients are mosaic, allowing some cells to express the mutant *MECP2,* while the others express the wild-type allele. XCI can be skewed preferentially so the mutant X chromosome is more or less expressed than the wild type, resulting in either a relatively more severe or milder presentation of RTT, respectively [98,99]. The mother of the children in the original Brazilian family presented no symptoms because her X-inactivation footprint skewed 95% of expression from the non-mutated chromosome [35]. Interestingly, several patients with the same mutation may exhibit a broad spectrum of clinical severity despite similar patterns of XCI in peripheral blood, potentially due to second gene mutations (modifiers) that alleviate or enhance the phenotypic outcome of *MECP2* mutation [100].

As an X-linked disorder, RTT was considered to be lethal in males. However, males with clinical features resembling classical RTT were reported even before the discovery of the causal gene. In 1999, Wan *et al*. [37] described the first mutation in *MECP2* in a male patient who died at one year of age from congenital neonatal encephalopathy. This discovery prompted the inclusion of boys in screening for *MECP2* mutations, allowing them to be classified into four categories: severe neonatal encephalopathy and infantile death, typical RTT, less severe neuropsychiatric phenotypes or *MECP2* duplication syndrome (table 2) [101–108].

**Table 2.** Males with *MECP2* mutations fall into four categories.

category	*MECP2* profile	features
severe neonatal encephalopathy and infantile death	*MECP2* mutation passed on by mildly symptomatic or asymptomatic mother	— spontaneously miscarried — if born, develop neonatal encephalopathy, respiratory arrest and seizures, death within 2 years
classical RTT	have at least partial Klinefelter's syndrome (XXY karyotype) or other somatic mosaicism	— symptoms similar to female RTT patients
less severe neuropsychiatric symptoms	*MECP2* mutations are less severe than those in female RTT patients	— symptoms are broad and overlap with features of Angelman syndrome (intellectual disability and motor abnormalities)
*MECP2* duplication syndrome	gain of *MECP2* dosage	— hypotonia, severe intellectual disability, recurrent lung infections, absent or limited speech and walking, seizures, motor spasticity and muscle stiffness — 50% die before age 25

## 4. Animal models inform *MECP2* function

*MECP2* appears across vertebrate evolution with strong conservation in its functional domains. *Drosophila* do not possess an orthologue to human *MECP2*, perhaps because the invertebrate genome is sparsely methylated [109]. In 2013, Pietri *et al*. isolated the first null mecp2^Q63*/Q63*^ zebrafish model, which does not recapitulate features of the human disorder [110,111]. Instead, the fish are viable and reproduce normally, but display minor motor abnormalities and a shortened lifespan, possibly due to immune deficiencies [112]. In 2017, Chen *et al*. [113] published the first analysis of *MECP2-*mutant cynomolgus monkeys, which exhibit decreased movement, social withdrawal, increased stereotypical behaviour and sleep abnormalities—symptoms common in RTT patients [113]. Overall, the monkeys exhibit CNS and transcriptome changes that are consistent with the human, in spite of being genetically heterogeneous [113]. Therefore, simian models may serve as a robust, albeit expensive, system to study sophisticated features of RTT in the future.

*Mecp2* mutant rodents recapitulate many hallmark symptoms of RTT and are the most widely accepted tool to study the disorder. An engineered null rat model of RTT (*Mecp2^ZFN^*) shows phenotypic and transcriptomic similarities to mouse models [114–116]. Although rats would have advantages for physiologic and preclinical testing, they are not used widely, perhaps due to their cost and the lack of genetic tools. Instead, two null mouse alleles are the primary models for Rett syndrome (table 3). The Bird laboratory developed a *Mecp2* mutant mouse model by engineering *lox*P sites flanking *Mecp2* exons 3 and 4 to create a ‘floxed’ conditional line [117]. This conditional-ready allele, called *Mecp2^tm1Bird^*, is hypomorphic, resulting in mild RTT-like symptoms due to a 50% reduction in MeCP2 expression [118,119]. A null mouse line lacking any protein product was created by crossing with a germline-deleting Cre driver (*Mecp2^tm1.1Bird^*/Y) [117]. Simultaneously, the Jaenisch lab designed a *Mecp2* mutant mouse by engineering *lox*P sites flanking exon 3, *Mecp2^tm1.1Jae^* [120]*.* A smaller *Mecp2* transcript and protein fragments are present in mutant brain; however, these mice show phenotypes similar to the null Bird allele.

**Table 3.** *Mecp2* mutant mouse models. The *Mecp2^tm1.1Bird^* and *Mecp2^tm1.1Jae^* alleles are the most commonly studied. Point mutation alleles are designed to mimic human RTT-causing mutations. Many conditional deletions have been created, but are not summarized here.

allele type	allele description	phenotypes	death (males)	reference
**null alleles**				
*Mecp2^tm1.1Bird^*	deletion of exon 3–4	stiff gait, reduced movement, hindlimb clasp, tremors, dishevelled fur, B6 underweight, 129 overweight	7–10 weeks	[79]
*Mecp2^tm1.1Jae^*	deletion of exon 3^a^	abnormal gait, hypoactive, tremors, mixed reports on weight	10 weeks	[82]
**point mutation alleles**				
*Mecp2^tm1Hzo^*	R308X; truncation	ataxia, tremors, dishevelled fur	>1 year	[89]
*Mecp2^tm1.1Jtc^*	R168X; truncation	hypoactive, hindlimb atrophy and clasping, breathing irregularities	12–14 weeks	[90,91]
*Mecp2^tm2.1Jae^*	S80A; missense	motor defects, slightly overweight	>1 year	[48]
*Mecp2^tm1Vnar^*	A140 V; missense	asymptomatic	>1 year	[92]
*Mecp2^tm1.1Meg^*	S421A; missense	asymptomatic	>1 year	[93]
*Mecp2^tm1.1Joez^*	T158A; missense	abnormal gait, hypoactive, hindlimb clasp, reduced weight	16 weeks	[94]
*Mecp2^tm1.1Mitoh^*	deletion of MeCP2 exon 2	asymptomatic but have placental defects	>1 year	[95]
*Mecp2^tm5.1Bird^*	R306C; missense	poor mobility, hindlimb clasping, tremors	18–25 weeks	[44]
*Mecp2^tm3Meg^*	T308A; missense	poor mobility, hindlimb clasping	>16 weeks	[45]
*Mecp2^tm1.1Dhy^*	deletion of MeCP2 exon 1	hypoactive, hindlimb clasping, excessive grooming	7–31 weeks	[96]
*Mecp2^tm4.1Bird^*	T158M; missense	poor mobility, hindlimb clasping, tremors	13 weeks	[97]
*Mecp2^tm6.1 Bird^*	R133C; missense	poor mobility, hindlimb clasping, tremors	42 weeks	[97]
*Mecp2^tm1.1Irsf^*	R255X; nonsense	breathing irregularities, heart defects	8–10 weeks	[98]
*Mecp2^tm3.1Joez^*	T158M; missense	abnormal gait, poor mobility, breathing irregularities, underweight	13 weeks	[99]
*Mecp2^tm4.1Joez^*	R106 W; missense	hypoactive, hindlimb clasping, underweight	10 weeks	[100]
**conditional alleles**				
*Mecp2^tm1Bird^*	floxed exons 3–4; hypomorphic	mild phenotype similar to *Mecp2^tm1.1Bird^* with delayed onset	as wild-type	[79]
*Mecp2^tm1Jae^*	floxed exon 3	no phenotype	as wild-type	[82]
*Mecp2^tm2Bird^*	floxed stop upstream exon 3	identical to *Mecp2^tm1.1Bird^*	10 weeks	[106]

^a^Some protein product retained.

Although RTT predominantly affects girls, the vast majority of published mouse studies take place in hemizygous *Mecp2* mutant male mice, because they present with a more consistent phenotype early in life. While female *Mecp2* mutant mice are more clinically relevant, random XCI in rodent females causes skewing of gene expression, resulting in large variations in phenotype presentation, making it difficult to separate which phenotypes arise through cell autonomous versus non-autonomous pathways [121]. The most commonly used male mouse models, *Mecp2^tm1.1Bird^* and *Mecp2^tm1.1Jae^*, consistently display overt phenotypes at four to six weeks of age, and die between 8 and 12 weeks of age [117,120]. Though *Mecp2* null mice are asymptomatic prior to four weeks of age, there are subtle but consistent defects in transcription, ionotropic receptor signalling, and neuronal responsiveness during late embryogenesis and the perinatal period [122–124]. At four weeks of age, null mice begin to develop uncoordinated gait, hypoactivity, tremors, hindlimb clasping reminiscent of the hand stereotypies seen in RTT patients, and irregular breathing. These phenotypes progress in severity until death. The soma and nuclei of *Mecp2* mutant neurons in the hippocampus, cerebral cortex and cerebellum are smaller and more densely packed than in wild-type littermates, probably giving rise to a smaller brain [120].

Curiously, body weight in *Mecp2* null mice differs depending on genetic background; male *Mecp2^tm1.1Bird^*/Y mice on a C57BL/6J background are substantially underweight by four weeks of age compared with wild-type littermates. On a 129S6/SvEvTac strain, males show a reverse effect: *Mecp2^tm1.1Bird^*/Y mice are the same weight as wild-type littermates until eight weeks of age, when they become significantly heavier than siblings [117]. Lastly, *Mecp2^tm1.1Bird^/*Y mice on a CD1 background weigh slightly less than wild type early in life, but reach a normal weight by six to seven weeks of age [125].

Heterozygous female mice also show no symptoms initially, but become hypoactive and start to hindlimb clasp at three months. By nine months of age, approximately 50% of heterozygous females develop the same phenotypes as their null counterparts, but some females remain asymptomatic at 1 year. Therefore, females have a lifespan that allows for reproduction, suggesting that the heterozygous condition can exhibit long-term stability, as seen in RTT [117]. Interestingly, heterozygous female mice on all genetic backgrounds, as well as female rats, become significantly overweight as they age [115,117,125], although humans with RTT have a wide range in body mass index [15].

In addition to deletion mouse models, several alleles have been engineered to recapitulate clinically relevant and common *MECP2* mutations in human patients. Mice with these alleles tend to display milder and later onset neurological phenotypes compared with the *Mecp2^tm1.1Bird^* null allele, and most do not recapitulate the entire disease phenotype (summarized in table 3) [70,71,126–138]. In an effort to distinguish the cause of specific phenotypes, *Mecp2* has also been conditionally deleted from different cell types and tissues [139–142]. A number of conditional deletions in different subsets of neurons highlight the essential role of MeCP2 in neuronal function, yet their milder presentation suggests additional roles outside the CNS (reviewed in [143]).

RTT patients show abnormal neuronal morphology, but not neuronal death, giving legitimacy to the possibility that defective MeCP2-deficient cells can recover [144]. In a landmark study, Guy *et al.* achieved symptom reversal in *Mecp2* mutant mice following the onset of motor dysfunction and neurological deficits. The *Mecp2^tm2Bird^* ‘*FloxedStop*’ allele, in which a *lox*P-STOP-Neo-*lox*P cassette was inserted into the intron upstream of endogenous *Mecp2* exon 3, was crossed with mice carrying a Cre recombinase fused to the oestrogen receptor (OR). Injection of tamoxifen (TM) caused Cre-ER to translocate to the nucleus and delete the FloxedStop cassette to reactivate the *Mecp2* gene [145]. *FloxedStop*/Y male mice behaved like *Mecp2^tm1.1Bird^*/Y mice, developed RTT-like symptoms between four and six weeks of age, and survived for approximately 10 weeks on average. TM injections in mice with advanced neurological symptoms and breathing abnormalities restored *Mecp2* expression to 80% of wild-type levels. Remarkably, restoration of MeCP2 expression reversed symptoms: neurological assessments and overall health improved and 80% of TM-treated animals survived beyond 30 weeks of age (end of study). Similarly, symptomatic *FloxedStop*/+ heterozygous female mice showed significant symptom improvement upon TM injection, including a reduction in body weight [145]. This pivotal finding showed that developmental absence of MeCP2 does not irreversibly damage neurons, instilling hope that symptom reversal is possible in RTT patients.

## 5. MeCP2 modulates transcription by bridging DNA with regulatory complexes

**Figure 3.**
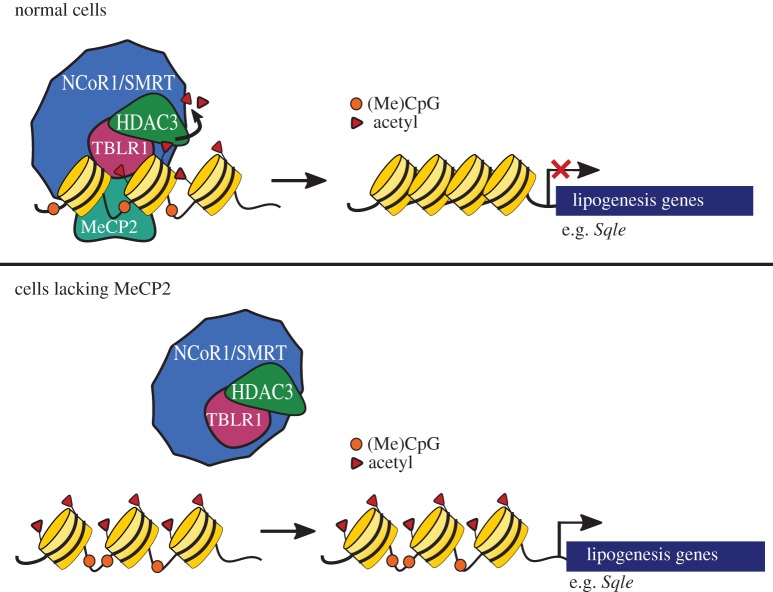
MeCP2 anchors the NCoR/SMRT to methylated DNA. In healthy cells, MeCP2 binds methylated CpG dinucleotides (orange circles) and recruits the NCoR1/SMRT-HDAC3 co-repressor complex to regulatory sites surrounding the target loci. HDAC3 removes acetylation marks from surrounding histones to compact chromatin and prevent transcription of target genes. In *Mecp2* mutant cells, the NCOR1/SMRT-HDAC3 complex cannot bind to methylated DNA resulting in an open chromatin state and increased transcription of genes. Known target genes of the complex in the liver include *Sqle* and other lipogenesis enzymes. Targets in the brain remain unknown.

In 2001, Kokura *et al.* [83] first demonstrated that MeCP2 binds to the NCoR1/SMRT co-repressor complex. NCoR1 and SMRT are highly homologous proteins that are recruited to chromatin to repress transcription by acting as a scaffold protein for other nuclear receptors, DNA-binding proteins and histone deacetylases [146]. One role of the NCoR1/SMRT complex is to recruit histone deacetylase 3 (HDAC3) to DNA, which removes histone acetyl marks, resulting in a closed chromatin state [147,148]. Other members of this complex include G protein pathway suppressor 2 (GPS2), transducin beta-like 1 (TBL1) and transducin beta-like 1 related (TBLR1) [149]. The interaction of MeCP2 with the NCoR1/SMRT complex was re-examined over a decade later when Lyst *et al.* [70] sought to identify binding partners of MeCP2. MeCP2 protein was purified from the brains of reporter mice with an enhanced green fluorescent protein (eGFP) inserted in the 3′ UTR of the *Mecp2* gene (*Mecp2^tm3.1Bird^*) and mass spectrometry was used to identify associated proteins. Remarkably, five of the seven MeCP2 protein interactors identified were subunits of the NCoR1/SMRT co-repressor complex [70]. Furthermore, this group found that the interaction between MeCP2 and the NCoR1/SMRT complex was facilitated by the NCoR1/SMRT interaction domain (NID) located at MeCP2 amino acids 285–309 [70,71]. MeCP2 binds to complex members TBL1 and TBLR1 at these residues via a WD40 domain, unlike other NCoR1/SMRT recruiters which interact with the NCoR1/SMRT scaffold proteins directly [150]. These findings have led to a model wherein MeCP2 serves as a bridge between methylated DNA and the corepressor complex, anchoring the NCoR1/SMRT complex to DNA via its NID and MBD, respectively. It is hypothesized that disruption of this bridge leads to histone hyperacetylation and an open chromatin state, thereby increasing the transcription of target genes (figure 3). Even so, transcriptional profiling studies using whole brain *Mecp2*/Y mice have not revealed dramatic gene expression changes [151,152], perhaps because the highly heterogeneous nature of the brain masks significant perturbations in subpopulations of cells.

RTT-causing missense mutations cluster in the MBD and NID, highlighting the importance of these two regions of *MECP2* in RTT pathology [153]. Within the NID is a mutational hotspot of MeCP2 between amino acids 302 and 306 [70,154]. R306C is one of the more common RTT mutations, and this allele was modelled in mice to determine the biological relevance of the NCoR1 corepressor complex in RTT (table 3) [68]. By six weeks of age, male *Mecp2^R306C^* mice develop tremors, hypoactivity, hindlimb clasping and motor activity defects. Fifty per cent of *Mecp2^R306^* mice fail to survive past 18-weeks of age and all die by 25 weeks [70]. The R306C mutant MeCP2 has an intact MBD, which binds to methylated heterochromatin. However, the mutation disables the NID, preventing MeCP2 from binding TBL1 or TBLR1, and thus the complex that contains NCoR1 and HDAC3, which disrupts MeCP2-mediated transcriptional repression. This suggests that the interruption of the MeCP2-NCoR1/SMRT complex interaction alone is capable of causing RTT-like phenotypes.

To test the primary role of the MeCP2-NCoR/SMRT1 interaction, the effect of a radically truncated MeCP2 protein was examined for symptom reversal in mice [155]. Mice were generated expressing a MeCP2 protein consisting of only the MBD, NID and short linker regions, while all other amino acid sequences of MeCP2 were removed. Remarkably, mice expressing this truncated protein develop only mild RTT-like symptoms and have a normal life span. Additionally, genetic re-activation or virus-mediated delivery of this minimal MeCP2 protein prevents or ameliorates symptoms in *Mecp2-*deficient pre-symptomatic or post-symptomatic mice, respectively. This suggests that a very important role of MeCP2 is to link DNA to the NCoR1/SMRT complex.

## 6. A mouse mutagenesis screen links MeCP2 to lipid metabolism

While *Mecp2* mutant mice are an excellent model for RTT, elimination of MeCP2 affects the expression of a profound number of pathways in the CNS, making it difficult to pinpoint which ones play a key role in pathology [77,89,90]. For this reason, Buchovecky *et al.* [156] employed an unbiased forward genetic suppressor screen in *Mecp2* null mice to identify mutations that alleviate RTT symptoms, which could be exploited as potential treatment targets. Genetic screening is a powerful tool to detect pathways involved in disease aetiology independent of *a priori* assumptions. Curiously, a dominant nonsense mutation was found in squalene epoxidase (*Sqle*), a monooxygenase and rate-limiting cholesterol biosynthesis enzyme, which requires an electron donor from mitochondria for its function [157]. This mutation in *Sqle* improved RTT-like motor phenotypes, overall health and lifespan in *Mecp2* null male mice [156]. This sparked the question: is cholesterol metabolism perturbed in *Mecp2* mutant mice?

**Figure 4.**
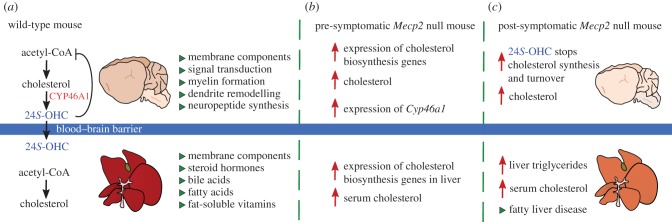
Cholesterol metabolism is perturbed in *Mecp2* mutant mice. (*a*) In the wild-type mouse, the brain produces cholesterol *in situ* as cholesterol cannot pass the blood–brain barrier (BBB). Acetyl-CoA enters the cholesterol biosynthesis pathway to make cholesterol which has many essential functions (green triangles). The enzyme CYP46A1 converts excess cholesterol into 24*S*-hydroxycholesterol (24*S*-OHC) for one-way egress across the BBB. The liver participates in cholesterol biosynthesis to provide cholesterol to other tissues in the body. (*b*) In pre-symptomatic *Mecp2* null mice (3–4 weeks old), increased expression of cholesterol biosynthesis genes in the brain leads to elevated brain cholesterol levels. Consequently, the expression of *Cyp46a1* is increased, indicating a heightened need for cholesterol turnover. Outside of the central nervous system, serum cholesterol is elevated, and expression of cholesterol biosynthesis genes is elevated in the liver. (*c*) In symptomatic *Mecp2* null mice (8–10 weeks old), the brain is smaller due to lack of *Mecp2*. Cholesterol biosynthesis decreases drastically in the brain, likely due to feedback from elevated 24*S*-OHC. Owing to decreased synthesis, brain cholesterol remains high, but not as high as at younger ages (smaller red arrow). Serum cholesterol and/or triglycerides may also be elevated, depending upon genetic background. Triglycerides accumulate in the liver, and fatty liver disease develops, as indicated by pale liver.

Cholesterol is a fundamental component of all cells [158,159]. While the liver is the primary manufacturer of cholesterol for the body, cholesterol is also a major component of the brain, where it functions in membrane trafficking, signal transduction, myelin formation, dendrite remodelling, neuropeptide formation and synaptogenesis [160]. Importantly, cholesterol cannot cross the blood–brain barrier (BBB), so any cholesterol that the brain requires must be synthesized *in situ* [161]. Therefore, the brain has complex and tightly regulated cholesterol synthesis pathways with multiple axes of self-regulation; too much or too little cholesterol is detrimental, and imparts negative consequences on cognition, memory and motor skills. To maintain homeostasis, cholesterol can be converted into the oxysterol 24(*S*)-hydroxycholesterol (24*S-*OHC) by the neuron-specific enzyme CYP46A1 (figure 4*a*) [162]. Oxysterols can pass lipophilic membranes more easily than cholesterol, allowing for one-way egress across the BBB and into the body [163].

Abnormalities in brain lipid homeostasis are associated with developmental disorders and diseases of ageing. Neimann–Pick type C patients accumulate cholesterol in the brain and peripheral tissues, and present with symptoms similar to RTT such as loss of acquired verbal skills and lack of motor coordination [164]. Patients with Smith–Lemli–Opitz syndrome develop microcephaly, cleft lip/palate, liver defects and autistic behaviours due to a deficiency in 7-dehydrocholesterol, allowing for the toxic build-up of cholesterol intermediates [165]. Furthermore, aberrant cholesterol homeostasis has been implicated in Fragile X syndrome [166], amyotrophic lateral sclerosis [167], Alzheimer's [168], Parkinson's [169] and Huntington's [170] diseases. However, lipid anomalies had never before been described in RTT and whether cholesterol played a primary role in the pathology of the disease was unknown.

Strikingly, Buchovecky *et al.* [156] found that cholesterol homeostasis is perturbed in the *Mecp2* null mouse brain. Pre-symptomatically, *Mecp2* null whole brain cholesterol is elevated, and *Cyp46a1* expression is increased 38% over wild-type levels, indicating a heightened need for cholesterol turnover in neurons [156] (figure 4*b*). Post-symptomatically, the overproduction of cholesterol decreases sterol synthesis *in Mecp2* null brains due to regulatory feedback [156,171] (figure 4*c*).

Notably, metabolic phenotypes differ in *Mecp2* null mice across genetic backgrounds. While both 129.*Mecp2^tm1.1Bird^* mice and B6.*Mecp2^tm1.1Jae^* mice show decreased sterol synthesis in the brain, elevated cholesterol and triglycerides in the serum and liver are observed only in 129.*Mecp2^tm1.1Bird^* mice [156]. Additionally, CD1.*Mecp2^tm1.1Bird^* mice have an increase in serum triglycerides with no corresponding change in serum cholesterol [125]. The C57BL/6 J inbred strain and the 129/Sv substrains manage peripheral cholesterol metabolism differently, presumably due to differences in the transport of cholesterol breakdown products [172]. Together, these data suggest that perturbed brain cholesterol synthesis is a common feature of *Mecp2* null mice; however, it is likely that genetic background contributes to the *Mecp2* null peripheral metabolic phenotype.

These differential findings in mice suggested that peripheral lipid markers would not be elevated in all patients. Consistently, only a subset of RTT patients exhibit increased serum cholesterol and/or triglycerides [15,16,173]. Outside of the CNS, sterols are the precursors of steroid hormones, bile acids and vitamin D. Interestingly, some RTT patients have bone abnormalities, severe gastrointestinal problems and biliary tract disorders [174–176]. As perturbations in lipid homeostasis may influence both neurological and non-neurological RTT symptoms, the measurement of lipid parameters may serve as a non-invasive and inexpensive biomarker to identify RTT patients who may benefit from treatments that target lipid metabolism.

## 7. MeCP2 regulates lipid metabolism with NCoR1/SMRT-HDAC3

Notably, the NCoR1/SMRT corepressor complex is an important component in regulating the diurnal control of energy metabolism. During the fasting cycle, the NCoR1/SMRT-HDAC3 complex maximally occupies hepatocyte chromatin, repressing the expression of genes involved in lipid synthesis and sequestration. However, during the feeding cycle, it releases chromatin, allowing for expression of its targeted loci [177]. This binding oscillation governs the switch between lipid and glucose utilization in hepatocytes. Mice with a liver-specific deletion of *Hdac3* display an increased expression of *de novo* lipogenesis enzymes due to a constitutively active transcriptional environment, resulting in fatty liver disease and elevated serum cholesterol [178,179]. Overall, the NCoR1/SMRT-HDAC3 complex orchestrates lipogenesis, and its disruption leads to perturbed lipid homeostasis. Therefore, it was expected that loss of MeCP2*,* the functional bridge between NCoR1/SMRT-HDAC3 and DNA, would lead to transcriptional dysregulation of lipogenesis and metabolic perturbations in mice. In support of this, *Sqle,* the suppressor of RTT-like phenotypes in mice, is a target of HDAC3, and its transcription increases approximately 170-fold in response to *Hdac3* liver deletion [156,178].

Indeed, Kyle *et al.* [180] showed that *Mecp2* mutant mice develop severe dyslipidaemia due to aberrant transcription of lipogenesis enzymes in the liver, because MeCP2 interacts with the NCoR1/SMRT-HDAC3 complex to repress lipogenic gene transcription. Loss of MeCP2 decreases HDAC3 binding to DNA, resulting in hyperacetylated histones, and increases transcription of numerous lipid-regulating genes. Interestingly, liver-specific deletion of *Mecp2* (B6.*Mecp2^tm1Bird^/*Y;*Alb-Cre*) also results in an increase in lipogenic enzyme transcription [180]. Similar to *Mecp2* null mice and a liver-specific deletion of *Hdac3*, these mice develop dyslipidaemia, fatty liver and metabolic disease. These results support a hepatocyte-autonomous role for *Mecp2* in co-ordinating repression of enzymes of the cholesterol and triglyceride biosynthesis pathways and show that loss of *Mecp2* from the liver is sufficient to cause metabolic disease in mice. Notably, background strain is unlikely to influence MeCP2's role in regulating liver lipid synthesis as both 129.*Mecp2^tm1.1Bird^* and B6.*Mecp2^tm1Bird^/*Y; *Alb-Cre* mice showed perturbed lipid metabolism.

## 8. Cholesterol-lowering statin drugs may be repurposed to treat RTT

Considering that cholesterol metabolism is perturbed in *Mecp2* mice, it was possible that pharmacological treatment with cholesterol-lowering drugs (statins) would improve symptoms. The primary mechanism of action for statin drugs is to competitively inhibit 3-hydroxy-3-methylglutaryl coenzyme A reductase (HMGCR), a rate-limiting step in cholesterol biosynthesis [181], effectively reducing the endogenous production of cholesterol. Owing to the prominent role of cholesterol in cardiovascular disease, statins are medically prescribed to prevent atherosclerosis. However, statins are associated with other disease-improving side-effects, such as reducing inflammation [182,183], and negative side-effects, such as muscle weakness [184] and acute memory loss [185]. Owing to their relative safety, recent studies have explored how statin drugs can be repurposed to treat non-cardiovascular diseases including Fragile X syndrome and neurofibromatosis Type I [186,187].

Remarkably, treatment with either fluvastatin or lovastatin improves subjective health scores, motor performance (measured by rotarod and open field activity) and increases lifespan in 129.*Mecp2^tm1.1Bird^* male and female mutant mice when compared with control mice receiving a vehicle [156]. Lovastatin is the more lipophilic of these statins, increasing the likelihood of crossing the BBB and entering the brain. Even so, treatment with either statin improves cholesterol homeostasis in the brains of *Mecp2* mutant mice, lowers serum cholesterol and ameliorates lipid accumulation in the liver. Interestingly, lovastatin does not have a strong therapeutic effect on B6.*Mecp2^tm1.1Bird^* male mice, supporting the idea that elevated peripheral lipids may be a biomarker for those patients who may respond to statin drugs [188]. Nevertheless, these findings support the idea that lipid metabolism, a pathway that has many opportunities for drug or dietary intervention, could be exploited for symptom improvement in a subset of RTT patients [156]. Importantly, these findings led to a phase 2 clinical trial for the pharmacological treatment of RTT with lovastatin (NCT02563860).

## 9. Implications for understanding and treating childhood neurological disorders

**Figure 5.**
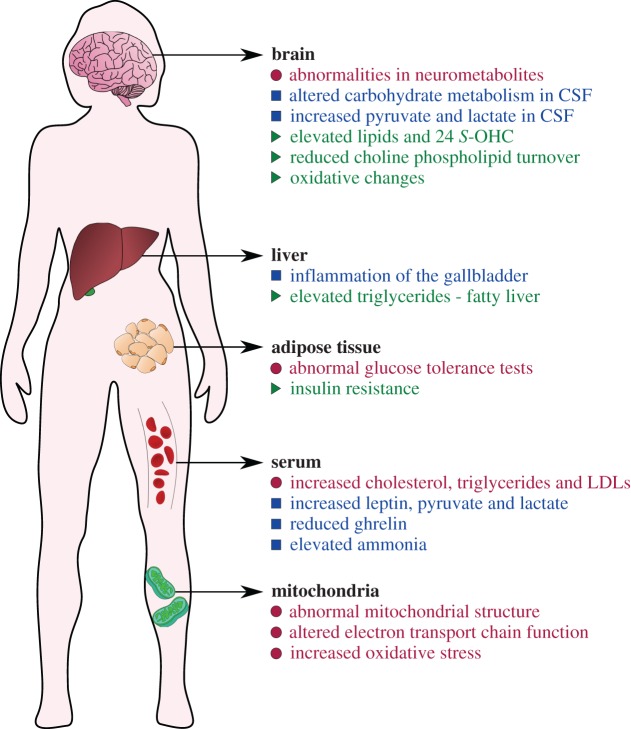
Metabolic components of Rett syndrome. Common metabolic disturbances observed in RTT patients (blue squares), *Mecp2-*mutant mouse models (green triangles) that may be a feature of human disease, or in both human and mouse (pink circles). Note that metabolic parameters may vary within the patient population or among different mouse strains: for example, hyperammonaemia was found in a subset of patients, but was dropped as a diagnostic criterion because it was not common. Such findings suggest that genetic variation may play a big role in the penetrance of all but key diagnostic features.

Rett syndrome remains a difficult disorder to understand and treat, largely because MeCP2 is central to the regulation of gene expression in many tissues and cell types. More than 60 years after the description of RTT as a metabolic disease of the nervous system, we have finally come to an understanding of some of the metabolic aspects of pathology (summarized in figure 5). Despite this, metabolic parameters vary greatly within the RTT patient population and among different mouse strains indicating that genetic variation may play a large role in the penetrance of metabolic symptoms.

In neurons, MeCP2 serves as a bridge between the NCoR1/SMRT-HDAC3 complex and DNA to facilitate transcriptional repression, and mutations that affect MeCP2 binding to the complex cause RTT. While both *Ncor1* and *Hdac3* null mice die as embryos, a neuron-specific deletion of *Hdac3* produces viable mice that gradually develop a phenotype very similar to that of *Mecp2* null mice including abnormal motor coordination, reduced sociability and cognitive defects [189]. These data suggest that at least a subset of RTT-like phenotypes in *Mecp2* mutant mice are caused by the loss of HDAC3 in neurons. Moreover, the role of the NCoR1/SMRT-HDAC3 in regulating lipogenesis in the *Mecp2* mouse liver raises interesting questions about the role of this complex in regulating lipids in the brain. Biochemistry studies have largely failed to find definitive gene regulatory targets, in part, because MeCP2 binds DNA at levels rivalling histone octamers [190]. Moreover, the brain is a complex organ with many different cell types in various stages of activity, making whole brain studies difficult to interpret. It is likely that at certain times and in specific cells, MeCP2 is required to anchor the NCoR1/SMRT-HDAC3 complex to DNA, regulating lipid production in some cells in the brain as in the liver.

As cholesterol synthesis is a resource-expensive process, current data suggest that newborn neurons must produce cholesterol in a cell-autonomous manner, but as they mature they outsource production to astrocytes (reviewed in [160,191]). Interestingly, wild-type neurons have abnormal dendritic morphology when co-cultured with MeCP2-deficient astrocytes, yet MeCP2-deficient neurons have normal dendritic morphology when co-cultured with wild-type astrocytes [192]. Moreover, deletion of MeCP2 from glial cells induces a RTT-like phenotype, and re-introducing MeCP2 to astrocytes of *Mecp2* null mice using GFAP-Cre improves symptoms and restores dendritic morphology [193,194]. These data could be explained by the hypothesis that at some point during development, cholesterol synthesis is reduced in a subset of neurons, and is provided instead by astrocytes; in the RTT brain, this downregulation does not occur, leading to lipid accumulation [160]. Certainly, this possibility may also explain the difference in timing of onset of symptoms in mice and humans, because the downregulation of cholesterol synthesis in neurons shortly precedes the onset of RTT-like symptoms in mice. New approaches provided by single-cell genomic technologies may provide definitive answers to this possibility.

Metabolic dysregulation in RTT and other diseases may impart substantial consequences on downstream systems. When one arm of metabolism is perturbed, it affects each connecting pathway, including glucose, lipid, amino acid, nitrogen and mitochondrial respiratory metabolism. Not surprisingly, both RTT patients and *Mecp2* mutant mice have abnormal responses to glucose tolerance tests, and mutant mice are also insulin-resistant [180,195]. Because *Mecp2* mice preferentially metabolize fat rather than glucose as their primary energy source, it is likely that other energy-sensing systems are affected. Specifically, energy metabolism affects the post-translational modification of proteins with O-linked *N*-acetylglucosamine (O-GlcNAc), a nutrient-driven epigenetic regulator [196,197]. The regulation of O-GlcNAc is required for many proteins involved in neurogenesis, and perturbation of O-GlcNAc protein modification has already been associated with many neurological diseases, including Alzheimer's [196,198]. Abnormal brain glucose-lipid homeostasis is also associated with oxidative stress in Alzheimer's [199]. RTT patients have increased oxidative burden and abnormal mitochondrial structure, while RTT animal models have defects in the mitochondrial respiratory chain [23,26,200–202] and oxidative changes in the brain [203]. Additionally, abnormal lipid metabolism is directly linked to inflammation [204], which is a component of pathology in both typical and atypical cases of RTT [205]. Notably, MeCP2 directly represses *Irak1* and downregulation of the NF-κβ pathway improves symptoms in *Mecp2* null mice [206].

The understanding of metabolic aspects of RTT pathology reveals potential therapeutic interventions. Statin drugs account for only one family of numerous metabolic modulators being developed to treat lipid accumulation or insulin resistance in Type II diabetes, which may be repurposed to treat RTT. Every year, new drug treatments are tested in *Mecp2* animal models that rescue different aspects of RTT phenotypes. For example, the treatment of *Mecp2* null mice with a protein-tyrosine phosphatase 1B (PTP1B) antagonist that was developed to treat Type II diabetes extended lifespan, decreased hindlimb clasping and improved motor performance [207]. Similarly, Trolox, a vitamin E derivative, normalized blood glucose levels, reduced oxidative stress and improved exploratory behaviour in *Mecp2* mice [208]. Additionally, insulin-like growth factor (IGF-1) partially rescued locomotor activity, respiratory function and heart rate in *Mecp2* mice [209,210]. In addition to statins, phase 2 clinical trials for RTT are in progress for a list of potential therapies including IGF-1 (NCT01777542), EPI-743 (NCT01822249), triheptanoin (NCT03059160) and NNZ-2566 (NCT01703533). However, despite this progress, no single treatment has yet fixed every phenotype in *Mecp2* mice or yet proved to effectively treat RTT symptoms.

While *MeCP2* has been largely studied in CNS development and maturation, some clinically significant aspects of RTT may arise independently of *MECP2* deficiency in the nervous system, and should be considered when planning any treatment strategy. Given the association between MeCP2 and HDAC3, non-specific HDAC inhibitors, commonly prescribed for seizure maintenance, should be approached cautiously as a treatment in RTT. Additionally, both RTT patients and *Mecp2* mutant mice present with metabolic syndrome [15,180], oxidative stress [27,29], cardiac defects [211,212], decreased bone density [174,213] and urological dysfunction [214,215]. As CNS-targeted gene therapy becomes a more realistic therapeutic approach, peripheral deficiency of MeCP2 must be considered more than ever as these symptoms are likely to persist following targeted genetic treatment to the brain.

Furthermore, mutations in members of the NCoR1/SMRT-HDAC3 complex should be examined for roles in other childhood neurological diseases. Already, mutations in other components of the complex, TBLR1 and TBL1, have been associated with autism, intellectual disability, Pierpont syndrome, a disorder characterized by developmental delay and abnormal fat distribution in the distal limbs, and West syndrome, a disorder with RTT-like features [216–219]. Notably, six of these mutations in TBLR1 mapped to the WD40 domain of the protein and disrupted MeCP2-binding [150]. Therefore, the transcriptional function of the NCoR1/SMRT complex could represent a shared mechanism for autism spectrum disorders and other neurological conditions. Comparing the transcriptome of mice with genetic deletions of *Mecp2* and members of the NCoR1/SMRT-HDAC3 complex may offer a comprehensive list of genes regulated by this interaction that can be studied to better understand disease pathology and exploited to develop potential treatments. Altogether, the progress in understanding the mechanistic basis for RTT pathology will continue to inform other neurological diseases and complex epigenetic mechanisms.

## Data accessibility

This article has no additional data.
